# Equol: A Microbiota Metabolite Able to Alleviate the Negative Effects of Zearalenone during In Vitro Culture of Ovine Preantral Follicles

**DOI:** 10.3390/toxins11110652

**Published:** 2019-11-09

**Authors:** Talyne Emilia Santos Silva, Danielle Cristina Calado de Brito, Naiza Arcângelo Ribeiro de Sá, Renato Felix da Silva, Anna Clara Accioly Ferreira, José Ytalo Gomes da Silva, Maria Izabel Florindo Guedes, Ana Paula Ribeiro Rodrigues, Regiane Rodrigues dos Santos, José Ricardo de Figueiredo

**Affiliations:** 1 Laboratory of Manipulation of Oocytes and Ovarian Pre-Antral Follicles (LAMOFOPA), Faculty of Veterinary Medicine, State University of Ceará, Fortaleza 60714-903, CE, Brazil; emiliataline@gmail.com (T.E.S.S.); daniellecalado@ymail.com (D.C.C.d.B.); naizarcangela@hotmail.com (N.A.R.d.S.); rsilva@gmail.com (R.F.d.S.); annaaccioly2014@gmail.com (A.C.A.F.); anapaula.ribeirorodrigues@gmail.com (A.P.R.R.); figueiredo.lamofopa@gmail.com (J.R.d.F.); 2 Laboratory of Biotechnology and Molecular Biology (LBBM), State University of Ceará, Fortaleza 60714-903, CE, Brazil; ytalogomes@hotmail.com.br (J.Y.G.d.S.); florinfg@uol.com.br (M.I.F.G.); 3 Schothorst Feed Research, 8218 NA Lelystad, The Netherlands

**Keywords:** xenoestrogens, mycotoxin, ovary, sheep, in vitro culture

## Abstract

The impact of zearalenone (ZEN) on female reproduction remains an issue, since its effects may differ among exposed cell types. Besides the use of decontaminants in animal diet, other approaches should be considered to minimise ZEN effects after exposure. Since the first organ in contact with ZEN is the gastrointestinal tract, we hypothesise that products of microbiota metabolism may play a role in ZEN detoxification. We aimed to evaluate the effect of 1 µmol/L ZEN and 1 µmol/L equol (a microbial metabolite), alone or in combination, on the survival and morphology of in vitro cultured ovarian preantral follicles. Ovaries from 12 sheep were collected at a local abattoir and fragmented, and the ovarian pieces were submitted to in vitro culture for three days in the presence or absence of the test compounds. The follicular morphology was impaired by ZEN, but equol could alleviate the observed degeneration rates. While ZEN decreased cell proliferation in primary and secondary follicles, as well as induced DNA double-strand breaks in primordial follicles, all these observations disappeared when equol was added to a culture medium containing ZEN. In the present culture conditions, equol was able to counteract the negative effects of ZEN on ovarian preantral follicles.

## 1. Introduction

Zearalenone (ZEN) is one of the most commonly found mycotoxins in food and feed, especially those based on corn, beet pulp, wheat, rice, barley, oats, and soybeans. A 10-year survey based on the analysis of 74,821 commodities from 100 countries demonstrated that 45% of the samples were contaminated with ZEN, at levels ranging from 1.53 ppm in rice up to 23.3 ppm in wheat [1]. This nonsteroidal oestrogenic mycotoxin is produced by the fungi of the genus *Fusarium* to control its reproduction. Due to structural and functional similarity to oestrogens, ZEN can also interact with animal cells and tissue structures, acting as an endocrine-disrupting chemical [2].

The negative impact of ZEN on fertility is well documented in humans [3,4] and farm animals, especially pigs and ruminants [5,6,7,8,9]. Most ZEN studies have focused on the action of this mycotoxin on cell lines [10,11], spermatozoa [8,12], or mature oocytes [6,13]. In a transgenerational study, Schoevers et al. [7] showed that immature oocytes, yet enclosed in preantral follicles, were sensitive to ZEN exposure, which affected follicular assembly, resulting in premature exhaustion of this follicle pool. Besides ZEN, diets usually contain phytoestrogens, which are plant-derived compounds with a structure similar to 17-β-oestradiol (E2), enabling them to induce (anti) oestrogenic effects depending on the dosage [14]. These phytoestrogens are divided into isoflavones, prenylflavonoids, coumestans, and lignans. Soybeans, alfalfa, and red clover are isoflavone-rich ingredients present in the diets of farm animals. Based on the fact that soybeans may also be contaminated with ZEN, its interaction with phytoestrogens should not be neglected. A biomonitoring study already showed the concomitant presence of the isoflavones genistein, daidzein, equol, and ZEN in serum and urine from pregnant women [15]. Unfortunately, these latter authors did not evaluate the possible interactions among these substances. It was recently demonstrated that genistein interacts with ZEN in vitro and, depending on the concentration range of both substances, the oestrogenic effect can be potentiated of inhibited [16]. Although, interaction studies between other phytoestrogens with mycotoxins are still lacking, one must bear in mind that ingested phytoestrogens are metabolised by reductase enzymes produced by the host microbiota. For example, soybeans and other legumes like alfalfa and red clover are rich in daidzein, which is converted to equol depending on the intestinal bacterial population of the animal [17]. Compared with its precursor daidzein, equol is more stable and more easily absorbable, and no other isoflavones shows stronger oestrogenic activity than equol [17]. Therefore, the interaction of ZEN with a microbiota product like equol should not be neglected in animals’ daily fed diets containing phytoestrogenic sources.

It has already been demonstrated that equol can be produced in several animal species, such as monkey [18,19], rat [18,19], pig [20,21], sheep [22], and human [19,23]. Equol also has a great affinity with oestrogen receptors, but depending on the dietary concentration, it may bring many beneficial health effects due to its antioxidant, antitumour, and anti-inflammatory properties [24]. Importantly, although both ZEN and equol are xenoestrogens and are usually originated from the same feedstuffs, they act differently. For instance, (i) equol preferentially binds oestrogen receptor (ER)-β, while ZEN has more affinity to ER-α; (ii) equol is a co-substrate to prostaglandin H synthase (PHS)-peroxidase stimulating PHS cyclooxygenase, while ZEN is an inhibitor [25]; (iii) equol inhibits the expression of the multidrug resistance protein ATP-binding cassette, subfamily G, member 2 (ABCG2 or BCRP [breast cancer resistance protein]) [26], while ZEN is an ABCG2 substrate [27]; and (iv) equol is not an antioxidant itself, but triggers cell signalling pathways to induce the synthesis of antioxidant enzymes [17], while ZEN induces oxidative stress [28]. Although these compounds are not often competing for the same oestrogen receptors, we hypothesise that the antioxidant and anti-inflammatory effects of equol may minimise the toxic effect of ZEN. Therefore, ovine ovarian fragments were in vitro cultured in the presence of ZEN, equol, or both, with the aim to evaluate the effect of equol on follicular morphology, development, and function.

## 2. Results

### 2.1. Morphology and Density of Preantral Follicles

During in vitro preantral follicle culture, morphological changes are observed according to the follicular development (e.g., primordial, primary, or secondary), and atresia can be detected by histological analysis. Ovarian pieces were cultured in vitro for three days to determine the effect of ZEN and equol, alone or in combination, on follicular development. Table 1 depicts the results obtained after morphological analysis. In vitro culture did not affect the percentage of morphologically normal primordial follicles when compared to non-cultured control.

**Figure 1:**
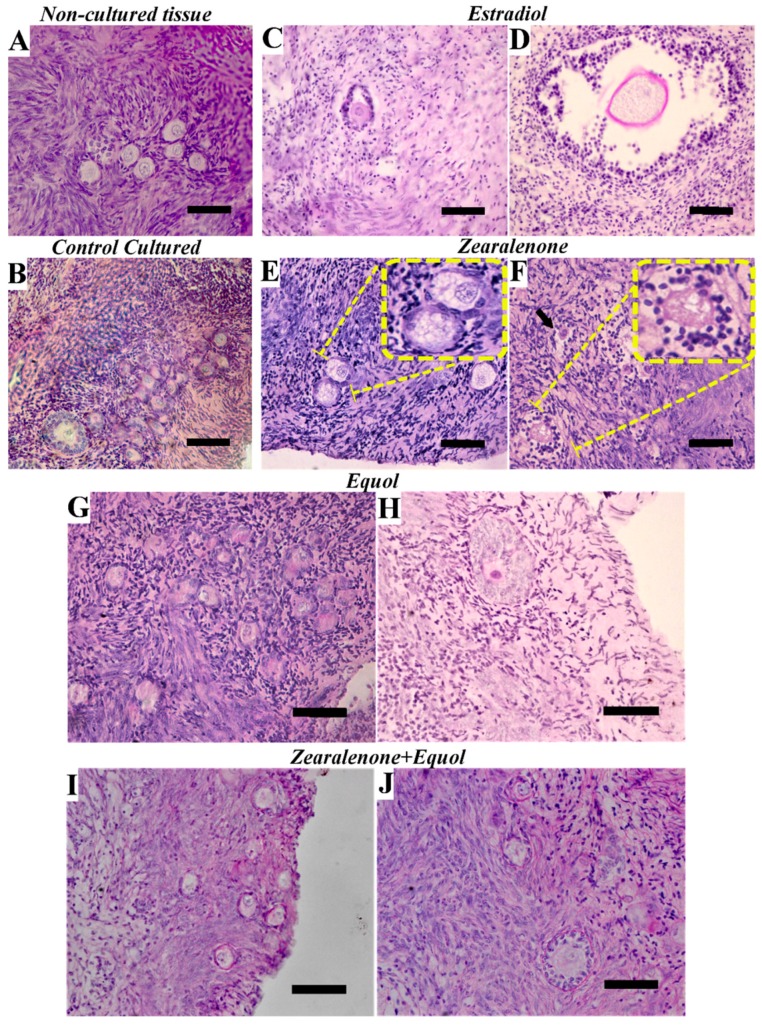
Representative images showing histological aspects of the preantral follicles before culture (**A**) primordial follicles, and after culture in the absence of xenoestrogens (**B**) follicles at different developmental stages, or in the presence of 3.12 μmol/L E2 (**C**,**D**) secondary follicles, 1 μmol/L ZEN (**E**) primordial follicles, (**F**) secondary follicle, 1 μmol/L Equol (**G**) primordial follicles, (**H**) secondary follicle), 1 μmol/L ZEN + 1 μmol/L Equol (**I**) primordial follicles, (**J**) secondary follicle with a primordial one). In panels E and F, inserts are given to show oocyte vacuolization in primordial and secondary follicles, respectively, after exposure to ZEN. Bar 50 µm. Staining: Haematoxylin-eosin.

Regarding primary and secondary follicles, their morphology was significantly impaired in all in vitro culture treatments, but with a prominently negative effect observed when ovarian fragments were exposed to E2. Although no differences were observed between ZEN and equol alone, a combination of ZEN with equol increased the percentage of morphologically normal secondary preantral follicles, being similar to untreated cultured tissue. Representative images of ovarian preantral follicles per class are given in Figure 1. It was remarkable that preantral follicles exposed to ZEN usually presented ooplasm vacuolisation.

During in vitro culture, it is expected that the pool of primordial follicles is activated for further development into primary and, subsequently, secondary follicles. The decrease in the density of primordial follicles with a concomitant increase in the density of secondary follicles was significant in the ovarian fragments cultured in non-supplemented medium, or in medium containing equol or ZEN + equol. The density of primordial follicles also decreased significantly during culture in the presence of E2, but without resulting in a significant increase in the density of primary or secondary follicles. When ovarian fragments were exposed to ZEN, a significant decrease of primordial follicle density was observed when compared to non-cultured control, followed by a significant increase in the density of primary follicles only (Table 2).

### 2.2. Nitrite and Malondialdehyde Levels in Culture Medium

None of the treatments affected nitrite and malondialdehyde (MDA) levels in the culture medium.

### 2.3. Oestradiol Levels in Culture Medium

Although oestradiol levels in the medium supplemented with 3.12 μmol/L E2 were significantly the highest when compared to all other treatments, no effect of the ZEN or equol was observed (*p* = 0.323). Oestradiol levels were measured as 35.3, 68.4, 48.5, and 27.1 ng/mL in the non-supplemented, ZEN, equol, and ZEN + equol group, respectively.

### 2.4. Quantitative RT-PCR

**Figure 2:**
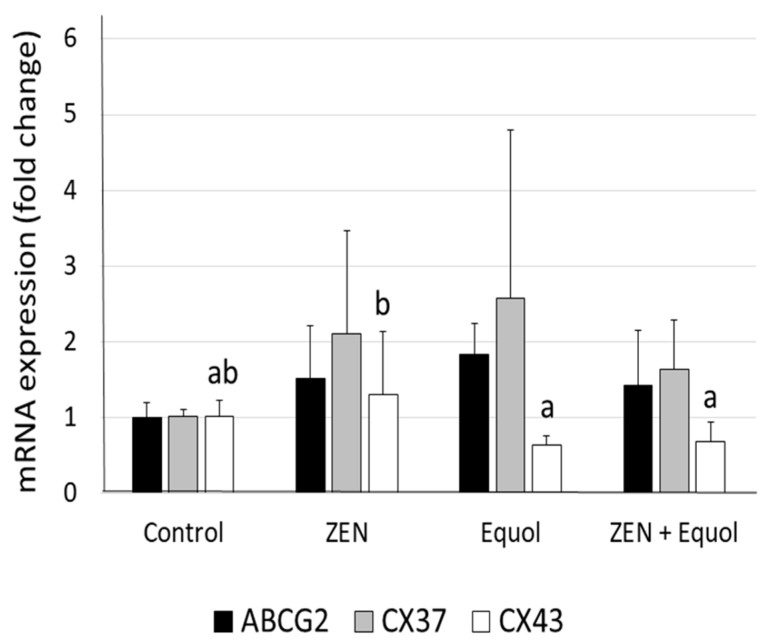
Mean (± SEM) levels of ABCG2, CX37 and CX43 in ovarian tissue cultured in the absence of xenoestrogens (control), or in the presence of 1 μmol/L ZEN, 1 μmol/L Equol or 1 μmol/L ZEN + 1 μmol/L Equol. a,b, different letters indicate significant difference among treatments (*p* ≤ 0.05).

No significant differences were observed in the mRNA expression of ABCG2, CX37, and CX43 when ZEN, equol, or their combinations were compared to the control group. However, a significant decrease in CX43 expression was observed in ovarian tissue exposed to equol or ZEN + equol when compared with the tissues exposed to ZEN only (Figure 2).

### 2.5. Immunohistochemistry

**Figure 3:**
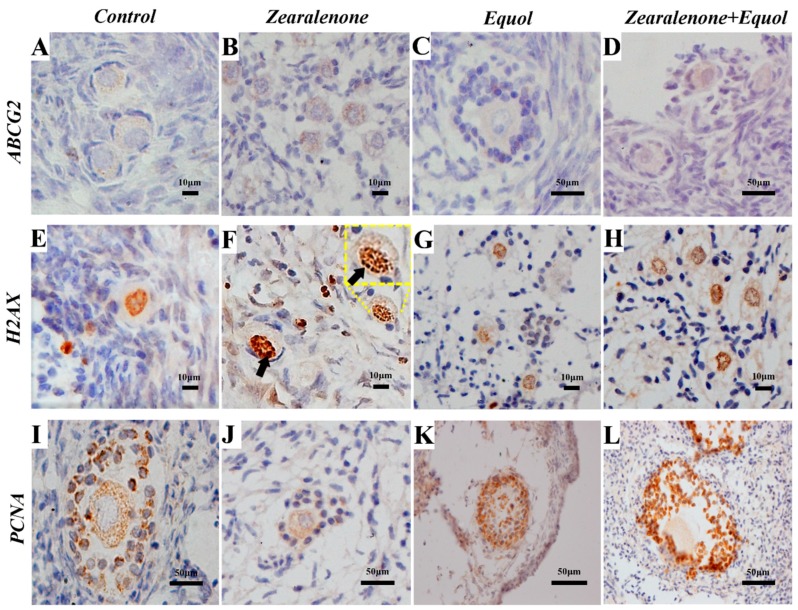
Representative images showing immunohistochemical staining for *ABCG2* (**A**–**D**), *H2AX* (**E**–**H**), and *PCNA* (**I**–**L**) in preantral follicles at different stages of development. The oolema of preantral follicles, regardless of the developmental stage and treatment, was similarly stained for *ABCG2* (**A**–**D**). The immunostaining of *H2AX* in the nucleus of preantral follicles is visible in panel F, where 3 or more foci per oocyte nucleus (see arrow). Decreased immunostaining was observed in granulosa cells from secondary follicles after culture in the presence of ZEN (**J**).

The percentages of follicles positively stained for ABCG2, H2AX, and PCNA at the different developmental stages (primordial, primary, and secondary) are detailed in Table 3. Representative images are given in Figure 3. All negative controls failed to show 3,3’-diaminobenzidine in chromogenic solution (DAB)-positive immunostaining, demonstrating the specificity of the markers.

#### 2.5.1. ABCG2 Protein Expression

A total of 1669 follicles (1258 primordial, 334 primary, and 77 secondary follicles) were investigated, and no differences were observed among the groups.

#### 2.5.2. H2AX Protein Expression

A total of 1609 follicles (921 primordial, 631 primary, and 57 secondary follicles) were investigated, and the results of immunohistochemistry showed that H2AX protein expression significantly increased in primordial follicles exposed to ZEN. No differences were observed between the control and equol groups. Furthermore, when ZEN was combined with equol, H2AX expression remained significantly higher than that observed in primordial follicles from the control group, but significantly lower than the rates of immunolabelled primordial follicles after exposure to ZEN.

#### 2.5.3. PCNA Protein Expression

A total of 1151 follicles (903 primordial, 215 primary, and 33 secondary follicles) were investigated, and the results of immunohistochemistry showed that proliferation of granulosa cells, as labelled by PCNA protein expression, was similar in primordial follicles, regardless of the treatment. However, exposure to ZEN significantly decreased the rate of granulosa cell proliferation in primary and secondary follicles, and this negative effect was counteracted when ZEN was combined with equol in the culture medium.

## 3. Discussion

In the present study, we demonstrated that even at low levels (1 µmol/L), ZEN can impair the in vitro development of preantral follicles, but this negative impact is minimised when these follicles are simultaneously exposed to ZEN and equol. Previous in vitro studies have showed that ZEN inhibits testosterone production by AM-10 Leydig cells (8 µmol/L) [29], increases the apoptosis rate in rat Sertoli cells (5 µmol/L) [30], affects gene expression in granulosa cells from mouse (30 µmol/L) and sows (10 µmol/L) [31], and impairs bovine oocyte maturation (1 mmol/L) [32] and porcine blastocyst rates (5 µmol/L) [33]. Although most of these concentrations are far higher than those tested in the present study, comparisons cannot be performed due to the differences of cell types and animal species. This also explains why we could not find any indication of oxidative stress or E2 modulation due to ZEN. Furthermore, chronic exposure cannot always be mimicked in vitro, since the period of culture may also affect cell viability, and in the present study the main focus was on the effect of ZEN in combination with equol.

ZEN is rapidly metabolised by the gastrointestinal tract after ingestion, producing some metabolites that are even more toxic, such as α-zearalenol [34]. Unfortunately, it is not possible to draw a parallel between in vitro and in vivo exposure, since there are no reference values for toxicological ZEN levels in biological matrices [35]. Levels of ZEN in plasma were determined in horses as 0.008 µmol/L–2.54 µg/L 24 h after being fed a diet containing 1 ppm ZEN [36], in cattle as 0.5 nmol/L–0.15 µg/L after being fed 0.66 ppm ZEN [37], and in women at levels up to 0.6 µmol/L–182.88 µg/L [38]. Concentrations of porcine follicular fluid were measured in a range of 15.2–54.8 µg/L (0.05–0.17 µmol/L) [39]. However, this last exposure level cannot be translated to preantral follicles because their oocytes are not yet surrounded by the antral cavity containing the follicular fluid. Importantly, it is known that preantral follicles seem to be more sensitive than antral ones when exposed to ZEN.

In a transgenerational study, Schoevers et al. [7] showed that exposure to ZEN via placenta and during lactation negatively affected the preantral population in porcine ovaries, and degeneration was mostly characterised by oocyte vacuolisation. At similar concentrations to those used in the present study, ZEN (25 µg/L; 0.08 µmol/L) also caused vacuolisation in canine granulosa cells [40]. Such vacuolisation was also remarkable in the present study after exposure to ZEN, but this process was minimised when ZEN was combined with equol in the culture medium. Vacuolisation has been linked to autophagy in oocytes [7] and hepatocytes [41] as a result of ZEN exposure. Autophagy was induced in IPEC-J2 cells via activation of the p38/MAPK activation in response to ZEN [42]. In Sertoli cells, ZEN induces autophagy by the inhibition of the PI3K/Akt/mTOR signalling pathway [43]. On the other hand, equol leads to the activation of this pathway in MCF-7 cells [44]. This could be one of the pathways involved in the interaction of these two xenoestrogens. Nevertheless, considering the complexity of the mode of action of such compounds and the different studied cell types, it is still not possible to determine all the factors involved in their interaction.

Equol is a metabolite of the host microbiota after degradation of daidzein, an isoflavone found in different feedstuffs like soybeans and other legumes like alfalfa. Mahalingam et al. [45] demonstrated that equol at a concentration of 100 μmol/L inhibits growth and induces atresia in mice antral follicle cultures in vitro. These authors also showed minor endocrine effects when follicles were cultured in the presence of 6 or 36 μmol/L of equol. At a concentration of 20 μmol/L, equol is able to induce apoptosis via caspase-3 cleavage [46]. However, when evaluating the effects of this metabolite, plasma levels should be considered. For instance, equol was found at levels of 0.5 mg/L (circa 2 µmol/L) in plasma from sheep fed red clover [22], 2.4 µmol/L in rats [18], 0.4 µmol/L in monkeys [18], 0.005 µmol/L in weaned piglets [20], and 0.05 µmol/L in sows [21]. In humans, equol plasma levels range from 0.4 to 1.2 μmol [23]. Importantly, plasma levels of this isoflavone will depend on dietary composition and intake, gender, age, and microbiota composition [23,47].

It was previously described that rats and pigs fed ZEN at concentrations of 250 and 10 mg/kg diet, respectively, could overcome ZEN toxicity when simultaneously fed a fibre-rich alfalfa meal [48]. These authors could not determine the exact compound counteracting the effect of ZEN, and they did not measure the levels of important phytoestrogens like equol. Most recently, it was shown that the flavonoid chrysin is capable of protecting mouse spermatozoa against the deleterious effects caused by dietary exposure (via gavage) to ZEN (40 mg/kg) [49]. Like equol, chrysin has antioxidant and anti-inflammatory properties. Although in the present study we could not find evidence of oxidative stress in the culture medium, in further studies we should evaluate the tissue itself together with markers of antioxidant capacity. Besides this, other pathways involving equol activity should be considered. Although ZEN has a higher affinity to ERα, it can also bind to ERβ, and when binding to ERα, ZEN leads to a stronger effect than via ERβ [50]. In preantral follicles, ERβ is expressed in granulosa cells, while the expression of ERα occurs in granulosa cells from antral follicles [51,52], indicating that the binding site of ZEN was preferably ERβ. The toxic effect of ZEN on normal prostate cells can be increased by blocking the activity of ERβ, indicating that this receptor may play a protective role against this mycotoxin [53]. Furthermore, ERβ is crucial for the subsequent differentiation of granulosa cells and follicular development [54]. Phytoestrogens like genistein [55] and resveratrol [56] are known to induce ERβ, and the same effect is expected for equol. This may explain the positive effect of equol on the proliferation of primary and secondary follicles, even when exposed to ZEN. ZEN can also induce cell cycle arrest, as observed by the decrease in PCNA labelling of proliferating rates of granulosa cells from primary and secondary follicles. Again, this effect was counteracted by equol. Although the present study was performed with reproductive cells, it should be pointed out that ERβ is also found in the intestines, the first organ exposed after oral contamination with ZEN via diet.

Only in primordial follicles, ZEN exposure increased the expression of the H2AX, a protein involved in DNA restoration during DNA double-strand breaks. DNA double-strand breaks can induce oocyte death or activate a programme of DNA repair [57]. It appears that in the present study, developing follicles (primary and secondary) were not selected for DNA integrity restoration, but only the pool of reserve (i.e., the primordial ones). The presence of equol in the culture medium, regardless of the presence of ZEN, did not induce the expression of the H2AX protein, indicating DNA protection. Except for the two-fold difference in the expression of CX37 when comparing ZEN with equol, no other difference was observed in gene regulation. Furthermore, such low fold change should not be considered biologically significant. This can be explained by the short-term in vitro culture for these types of cells [58], as well as the tested concentrations of the substances. For instance, ABCG2 is usually upregulated by ZEN [27] and downregulated by equol [26], but no differences were observed in the present study.

In conclusion, equol may alleviate the toxic effect of ZEN by counteracting oocyte vacuolisation and supporting granulosa cell proliferation and subsequent follicular development. It seems that different pathways play a role, and this needs to be understood. Equol production will depend on the host microbiota, meaning that complex and well-functioning microbiota will help the host defense against ZEN. The microbial population may vary among individuals, which may explain the individual variation on the effects of ZEN toxicity within different animal species. Daidzein-rich diets as a source of substrate for microbial equol production might not be sufficient, and gut modulation with probiotics able to degrade daidzein into equol would improve gut health and, subsequently, animal reproduction. The present study was performed in vitro at two fixed concentrations of both xenoestrogens as a proof of principle. To confirm the present findings, in vivo studies are still needed.

## 4. Materials and Methods

### 4.1. Test Compounds

Unless mentioned otherwise, substances used in the present experiment were purchased from Chem Cruz (Santa Cruz Biotechnology, Inc., Dallas, TX, USA) and stock solutions were prepared using dimethyl sulfoxide (DMSO) as a solvent. The final DMSO concentration added to the culture medium was 0.1%. The culture medium and 17β-estradiol were purchased from Sigma Chemical Co. (St. Louis, MO, USA).

### 4.2. Animal Ethics Statement

This study was approved and conducted according to the Animal Management and Ethical Regulation Committee of the State University of Ceará (N° 9433833/2018). Ovarian tissues were collected in a commercial abattoir.

### 4.3. Ovarian Tissue Collection

Sheep ovarian pairs (*n* = 12) were collected from 12 adult sheep in a local abattoir immediately after slaughter. After collection, ovaries were washed one time in alcohol 70% for 10 s, followed by two washes in Minimum Essential Medium (MEM) supplemented with N-2-hydroxyethylpiperazine-N-ethanesulfonic acid (HEPES) (MEM-HEPES), penicillin (100 μg/mL) and streptomycin (100 μg/mL). Then, the ovaries were stored in sterile tubes with the same medium and transported to the laboratory at 4 °C within one hour.

### 4.4. Experimental Design

In an initial trial, each of nine sheep ovarian pairs was divided into six fragments of 3 × 3 × 1 mm, and randomly assigned to the different experimental conditions. Of these, one fragment was randomly selected as fresh control, and the remaining eight fragments were in vitro cultured in non-treated culture medium, or in culture medium supplemented with estradiol (E2), zearalenone (ZEN), equol, or combinations of ZEN and equol (ZEN + equol). All supplements were used at a concentration of 1 µm/L, except for the E2 that was added at a concentration of 3.12 μmol/L. The exposure to E2 during in vitro culture was based on its structural similarity with phytoestrogens, and the high tested concentration was chosen due to its known negative effect in oocytes [59]. Concentrations of the other test compounds were selected based on plasma levels of equol encountered in ovine (1 μmol/L) [22] and the previously tested level of ZEN in combination with xenoestrogen (1 μmol/L) [16]. After in vitro culture, ovarian fragments were fixed for histological analysis (morphology, follicular development and stromal density) and culture media were separately analysed for E2 levels, as well as for oxidative stress using nitrite and malondialdehyde (MDA) as markers.

Simultaneously, three pairs of sheep ovaries were divided into 14 fragments of 3 × 3 × 1 mm each, and randomly assigned to the different experimental conditions. The 14 fragments were in vitro cultured in non-treated culture medium, or in culture medium supplemented with ZEN, equol, or ZEN + equol (two fragments per treatment). All supplements were used at a concentration of 1 µm/L. After in vitro culture, the fragments were assessed via immunohistochemistry and quantitative real-time polymerase chain reaction (qRT-PCR).

### 4.5. In Vitro Culture

Fragments of ovarian cortex were individually in vitro cultured in 24-well plates containing 1 mL of culture medium each. Culture medium consisted of α-MEM supplemented with 10 μg/mL insulin, 5.5 μg/mL transferrin, 5 ng/mL selenium, 2 mg/L glutamine, 2 mg/L hypoxanthine, and 1.25 mg/mL bovine serum albumin (BSA). According the experimental group, ovarian fragments were cultured in the absence or presence of the test compounds, as mentioned above. Dimethyl sulfoxide (DMSO) at a concentration of 0.1% was used as dilution vehicle and was added as well in the non-supplemented culture medium. The fragments were then cultured for three days at 38 °C in humidified air with a 5% CO_2_ atmosphere.

### 4.6. Histological Analysis

The ovarian fragments were fixed in 4% paraformaldehyde and then dehydrated at increasing ethanol concentrations. Subsequently, these fragments were embedded in paraffin and cut into 7 μm sections, mounted under glass slides and stained with Periodic Acid-Schiff (PAS) and haematoxylin. For morphological evaluation, slides were examined under an optical microscope (Nikon, Tokyo, Japan) at 400× magnification. The analysed preantral follicles were classified into primordial (oocyte surrounded by a layer of squamous granulosa cells), primary (oocyte surrounded by a layer of cubic granulosa cells), or secondary (oocyte surrounded by two or more layers of cubic granulosa cells) [60]. In order to avoid double counting, follicles were counted only in sections where their oocyte nucleus was observed. Follicles were classified as morphologically normal or degenerated considering the following characteristics: absence or presence of pycnotic bodies, cytoplasmic retraction and granulosa cell organization, as described by Santos et al. [61].

### 4.7. Density of Preantral Follicles

The density of preantral follicles, per development group or as total, was calculated as the total number of follicles, per class, divided by the total tissue volume and expressed as the number of follicles/mm^3^ of ovarian tissue [62]. The volume of the analysed ovarian sections was calculated by summing the number of sections of area multiplied by the thickness of each section.

### 4.8. Nitrite and Malondialdehyde Quantification in Culture Medium

Exposure to ZEN may result in oxidative stress and cell membrane damage, which can be characterized by an increase in nitric oxide production and lipid peroxidation [63]. Such an effect can be determined in cell culture suspensions, where an increase in nitrite levels are indicative of oxidative stress and membrane lipid peroxidation will result in increased malondialdehyde (MDA) levels. For the evaluation of Nitrite and MDA levels, 200 μL of culture medium from each treatment were collected and stored in a freezer −80 °C for later measurement. For Nitrite levels, a protocol already established by Green et al. [64] was applied. In summary, 100μL of Griess reactive (1% sulfanylamide, 0.1% N-(1-naphthyl)-ethylenediamine hydrochloride, H_3_PO_4_ in 1%, 1:1:1:1 distilled water) was added to 100 μL of the culture medium. Then the samples were incubated at room temperature for 10 min. The standard curve was elaborated with various sodium Nitrite concentrations (ranging from 0.25 to 2.378 μM), and the samples were evaluated at a wavelength of 560 nm using a microplate reader (Metertech Inc., Taipei, Taiwan). The blank (negative control) was prepared by adding 100 μL of Griess reactive to 100 μL of the base medium.

To assess MDA levels, the TBARS (thiobarbituric acid reactive substances) method was used. For this, 100 μL of the culture medium was used and incubated in a water bath at 37 °C for 1 h. Subsequently, 80 μL of 35% perchloric acid was added and centrifuged at 14,000 rpm at 4 °C for 10 min. Thereafter, 50 μL of thiobarbituric acid was added to the supernatant and the mixture was incubated at 100 °C for 30 min [65]. After cooling to room temperature, the measurement was performed by absorbance at a wavelength of 532 nm in a microplate reader (Metertech Inc., Taipei, Taiwan).

### 4.9. Estradiol Dosage

To measure estradiol levels in the culture media, the enzyme-linked fluorescent assay (ELFA) was applied according to the manufacturer’s instructions (VIDAS^®^ Estradiol II, refª 30431 bioMérieux SA, 376 Chemin de L’Orme, 69280 Marcy-l’Etoile, France). The fluorescence intensity emission was recorded at 450 nm, and the fluorescence signal values were inversely proportional to the antigen concentration present in the samples. The coefficient of variation was of 7.5% and the lower limit of detection was of 9 pg/mL.

### 4.10. Immunohistochemistry

It was previously reported that expression of the multidrug resistance protein ATP-binding cassette, subfamily G, member 2 (ABCG2 or BCRP [breast cancer resistance protein]) can be modulated by both equol and ZEN [26,27]. Signs of DNA repair due stress were detected by H2A histone family member X (H2AX) labelling [57]. Proliferation rate of granulosa cells per follicle was determined by proliferating cell nuclear antigen (PCNA) labelling. Paraffin sections were deparaffinized with xylene (Xylene, Sigma-Aldrich, St. Louis, MO, USA) and rehydrated in alcohol series. The endogenous peroxidase activity was blocked with 3% H_2_O_2_ diluted in methanol. A demasking step was performed for 75 min at 98 °C with citrate buffer and 20% Triton X100 before sections were subjected to antigen retrieval step. Antigen retrieval steps, antibody dilutions and incubation conditions are summarized in Table 1. In brief, slides were incubated for 30 min at room temperature with anti-PCNA rabbit polyclonal primary antibody (Abcam Inc., Cambridge, MA, USA), anti-H2AX (Abcam Inc.), or anti-ABCG2 (Novus Biologicals, Centennial, CO, USA). Subsequently, slides were incubated for 30 min in the Goat anti-rabbit IgG secondary antibody (1: 200 dilution; Abcam) (Table 4). The slides were then incubated for 30 min with the avidin-biotin enzyme complex (ABC; Vector laboratories, Burlingame, CA, USA) to react with 3,3’-diaminobenzidine in chromogenic solution (DAB; Dako Inc., Santa Clara, CA, USA). Hematoxylin and Scott’s solution were used for counterstaining. Negative controls were performed under the same conditions, but in the absence of the primary antibody. The following positive controls were used: spleen for PCNA and poisoned heart for H2AX. For quantitative analysis, follicles were considered PCNA-positive when at least one granulosa cell was stained and were considered as belonging to the growing pool (primary or secondary follicles), H2AX-positive when the oocyte was immunostained; ABCG2-positive when oocytes and or granulosa cells were immunostained.

### 4.11. Ribonucleic Acid (RNA) Extraction and Quantitative Real-Time PCR

Besides ABCG2 modulation by ZEN, this mycotoxin may also impair cellular gap junctions [66]. Therefore, mRNA expression of ABCG2 and gap junction proteins present in ovarian follicles, e.g., Connexin 37 (CX37) and -43 (CX43) was determined. For evaluation of gene expression levels for ABCG2, CX37 and CX43, the ovarian fragments were submitted to RNA extraction with TRIzol^®^ Plus RNA Purification Kit (Invitrogen, São Paulo, SP, Brazil). Isolated RNA preparations were treated with DNase I and Pure Link ™ Mini RNA Kit (Invitrogen, Sao Paulo, SP, Brazil). Complementary DNA (cDNA) was synthesized from RNA isolated using the SuperscriptTM II RNase H Reverse Transcriptase (Invitrogen, Sao Paulo, Brazil). The qPCR reactions had a final volume of 20 μL and contained the following components: 1 μL cDNA as template in 7.5 μL SYBR Green Master Mix (PE Applied Biosystems, Foster City, CA, USA), 5.5 μL of ultrapure water and 0.5 μL of each primer. Primers were designed to amplify ABCG2, CX37 and CX43 mRNA levels (Table 5). The reference genes used as endogenous control for normalization, gene expression evaluation and gene expression stability in all samples were glyceraldehyde-3-phosphate dehydrogenase (GAPDH) and β-actin (ACTB). Primer specificity and amplification efficiency were verified for each gene. Expression stability of these genes was analysed using BestKeeper software. The thermal cycle profile for the first qRT-PCR cycle was as follows: initial denaturation and polymerase activation for 15 min at 94 °C, followed by 40 cycles of 15 s at 94 °C, 30s at 60 °C and 45 s at 72 °C. The final extension was 10 min at 72 °C. All reactions were performed on a real-time PCR Mastercycler (Eppendorf, Germany, Hamburg, Germany, USA). Data were analysed using the efficiency-corrected Delta-Delta-Ct method [67]. The fold-change values of the genes of interest were normalized using the geometric mean of the fold-change values of two housekeeping genes.

### 4.12. Statistical Analysis

The ovarian fragments were randomly assigned to treatment conditions prior each experiment. Statistical analysis was conducted with the GenStat statistical software (GenStat for Windows 18th Edition, VSN International, Hemel, Hempstead, UK; https://www.vsni.co.uk/downloads/genstat/). Data were compared with ANOVA. The null hypothesis was that there was no treatment effect on the response parameter. Treatment means were compared according Fishers’ LSD (for a two-sided test and *p* ≤ 0.05) after a significant treatment effect was confirmed by ANOVA. Data are presented as mean ± SEM. The *p*-value of the statistical model is given per response parameter. Effects with *p* ≤ 0.05 were considered to be statistically significant.

## Figures and Tables

**Table 1:** Mean (±SEM) percentages of morphologically normal preantral follicles cultured or not in the presence of zearalenone alone or combined with equol.

Treatments	Morphologically Normal Preantral Follicles (%)					
	Primordial		Primary		Secondary	
Non cultured control	76.3 ± 12.6	b	78.0 ± 11.6	c	97.1 ± 6.39	d
Cultured control	64.4 ± 10.4	b	58.3 ± 16.5	b	74.7 ± 22.5	c
Estradiol	52.0 ± 12.3	a	36.2 ± 15.7	a	13.6 ± 19.3	a
ZEN	51.8 ± 11.2	a	45.1 ± 19.4	ab	50.6 ± 23.7	b
Equol	65.6 ± 14.6	b	48.1 ± 19.8	ab	53.6 ± 15.9	bc
ZEN+Equol	66.6 ± 14.2	b	60.2 ± 20.9	b	65.7 ± 25.4	bc
						
*p*-values	0.001		<0.001		<0.001	

a–d different letters indicate significant difference among treatments (*p* ≤ 0.05).

**Table 2:** Mean (±SEM) density of preantral follicles cultured or not in the presence of zearalenone alone or combined with equol.

Treatments	Density of Preantral Follicles (Follicles/mm^3^)					
	Primordial		Primary		Secondary	
Non cultured control	237 ± 53	b	53 ± 18	a	3.2 ± 2.1	a
Cultured control	154 ± 47	a	69 ± 40	a	10.9 ± 3.1	bc
Estradiol	93 ± 48	a	55 ± 27	a	7.6 ± 6.7	ab
ZEN	151 ± 98	a	127 ± 59	b	8.5 ± 7.7	ab
Equol	115 ± 78	a	58 ± 33	a	18.6 ± 10.8	c
ZEN+Equol	81 ± 48	a	81 ± 33	a	12.1 ± 8.3	bc
						
*p*-values	0.001		0.002		0.042	

a–c different letters indicate significant difference among treatments (*p* ≤ 0.05).

**Table 3:** Summary of the expression of ABCG2, H2AX, and PCNA protein in ovarian tissue.

Factors	Control *n* (% stained)	ZEN *n* (% stained)	Equol *n* (% stained)	ZEN + Equol *n* (mean % ± SEM)	*p*-Values
*ABCG2*					
Primordial follicles	251/319	365/436	232/309	162/194	
Oocytes (cytoplasm)	76.3 ± 6.0	83.2 ± 1.0	74.8 ± 1.0	83.3 ± 3.2	0.056
Primary follicles	80/101	96/110	46/58	52/65	
Oocytes (cytoplasm)	79.3 ± 3.7	84.0 ± 5.4	77.5 ± 8.7	80.6 ± 3.1	0.661
Secondary follicles	26/29	16/18	14/15	13/15	
Oocytes (cytoplasm)	86.0 ± 10.0	90.3 ± 5.8	88.9 ± 11.0	86.1 ± 7.4	0.937
*H2AX*					
Primordial follicles	132/321	141/228	72/162	108/210	
Oocytes	40.7 ± 5.5 a	62.7 ± 4.2 c	44.4 ± 1.2 a	51.5 ± 9.4 b	*p* < 0.001
Primary follicles	89/223	75/171	53/123	43/114	
Oocytes	38.4 ± 0.9	42.8 ± 3.4	38.5 ± 4.6	42.5 ± 6.3	0.514
Secondary follicles	6/22	6/18	5/14	5/12	
Oocytes	31.3 ± 3.3	46.7 ± 3.5	33.3 ± 2.9	33.3 ± 3.4	0.477
*PCNA*					
Primordial follicles	97/235	144/336	69/204	58/168	
Granulosa cells/follicle ^1^	9.3 ± 1.9	7.6 ± 1.8	13.1 ± 5.3	8.6 ± 3.4	0.248
Primary follicles	66/86	53/73	57/83	52/63	
Granulosa cells/follicle ^1^	37.4 ± 6.4 a	18.2 ± 1.8 b	28.4 ± 13.2 ab	36.2 ± 3.5 a	*p* < 0.01
Secondary follicles	17/17	15/15	18/18	13/13	
Granulosa cells/follicle ^1^	75.5 ± 4.8 a	55.1 ± 9.3 b	81.7 ± 4.5 a	77.2 ± 7.0 a	*p* < 0.001

^1^ Mean percentage of positive granulosa cells per follicle. a–c different letters indicate significant difference among treatments (*p* ≤ 0.05).

**Table 4:** Antibodies dilutions used in the present study.

Antigen	Antibody	Dilution	Incubation
*ABCG2*	Rabbit monoclonal primary antibody	1:50	4° C O/N
*H2AX*	Mouse monoclonal gamma primary antibody	1:100	4° C O/N
*PCNA*	Rabbit polyclonal primary antibody	1:25	4° C O/N

**Table 5:** Primers used for the quantification of genes of interest and housekeeping gene expression.

Genes	GenBank Accession n^o^.	Primer	Sequence
*ACTB*	XM_018039831.1	Forward Reverse	CTTCCTTCTTGGGTATGGA ACGGATGTCAACGTCACACT
*GAPDH*	XM_018049688.1	Forward Reverse	ATGCCTCCTGCACCACCA AGTCCCTCCCACGATGCCA
*ABCG2*	XM_018049131	Forward Reverse	CGGCATTCCAGAGACAACCT CGGCATTCCAGAGACAACCT
*CX37*	AY745977.1	Forward Reverse	CGACGAGCAGTCGGATTT AGATGACATGGCCCAGGTAG
*CX43*	AY074716.1	Forward Reverse	ATGAGCAGTCTGCCTTTCGT TCTGCTTCAAGTGCATGTCC

## Abbreviations

ABCG2/BCRP	ATP-binding cassette, subfamily G, member 2/breast cancer resistance protein
ACTB	Beta actin
ANOVA	Analysis of variance
BSA	Bovine serum albumin
cDNA	Complementary deoxyriboNucleic Acid
CX37	Connexin 37
CX43	Connexin 43
DAB	3,3’-diaminobenzidine in chromogenic solution
DMSO	Dimethyl sulfoxide
DNA	DeoxyriboNucleic Acid
E2	17-β-oestradiol
ELFA	Enzyme-linked fluorescent assay
ER-α	Oestrogen receptor alpha
ER-β	Oestrogen receptor beta
GAPDH	Glyceraldehyde 3-phosphate dehydrogenase
H2AX	H2A histone family member X
HEPES	N-2-hydroxyethylpiperazine-N-ethanesulfonic acid
H_2_O_2_	Hydrogen peroxide
H_3_PO_4_	Phosphoric acid
IgG	Immunoglobulin G
IPEC-J2	Intestinal Porcine Epithelial Cell line-J2
LSD	Least significant difference
MA-10 Leydig cells	Mouse Leydig tumor cell line
MCF-7	Breast cancer cells (Michigan Cancer Foundation-7)
MDA	Malondialdehyde
MEM	Minimum essential medium
PAS	Periodic Acid-Schiff
PCNA	Proliferating cell nuclear antigen
PHS	Prostaglandin H synthase
PI3K/Akt/mTOR	Phosphatidylinositol 3-kinase/serine-threonine protein kinase/mammalian target of rapamycin
P38/MAPK	p38 mitogen-activated protein kinase
qRT-PCR	Quantitative real-time polymerase chain reaction
RNA	Ribonucleic acid
TBARS	Thiobarbituric acid reactive substances
ZEN	Zearalenone
